# Impact of perceived environmental legacy on residents’ pro-environmental behaviors in large-scale sport events: a moderation analysis of authenticity

**DOI:** 10.3389/fspor.2025.1645623

**Published:** 2025-10-31

**Authors:** Jiexin Chen, Ji Wu, Yigang Wu

**Affiliations:** ^1^ School of Economics and Management, Shanghai University of Sport, Shanghai, China; ^2^ School of Physical Education, Shanghai University of Sport, Shanghai, China

**Keywords:** perceived environmental legacy, pro-environmental behaviors, authenticity, large-scale sport event, Norm Activation Model

## Abstract

**Introduction:**

Prior research has demonstrated that residents' perceptions of environmental legacy (PEL) following large-scale events yield several positive outcomes, such as subjective well-being and support of future events. However, the role of PEL in shaping residents' pro-environmental behaviors (PEBs) remains underexplored. Drawing on the Norm Activation Model, this study investigated how PEL is associated with PEBs through the mediation of perceived fit (PFT) and the moderation of authenticity evaluation (AUTH).

**Methods:**

Data were collected via an online survey administered to 823 residents of Hangzhou, one year after the 2023 Hangzhou Asian Games. Structural equation modeling revealed that PFT fully mediated the relationship between PEL and PEBs.

**Results:**

Latent moderated structural modeling showed that AUTH strengthened the direct relationship between PEL and PEBs.

**Discussion:**

The study advances event legacy literature by identifying the psychological pathways associated with the activation of PEBs, which are mediated by PFT and moderated by AUTH. For policymakers and event organizers, the findings underscore the importance of designing environmental legacies that are both authentic and culturally resonant to maximize long-term behavioral impacts. To enhance residents' PEBs, stakeholders in large-scale sports events should prioritize the authenticity and value-fit of their environmental strategies.

## 1 Introduction

Large-scale sport events, such as the Olympic Games, FIFA World Cup, and Asian Games, serve as globally influential platforms designed to deliver social and economic benefits for host communities. However, a growing stream of research highlights that supporters of large-scale sport events often overlook the environmental harms imposed on these communities during the period of event planning and hosting (1–4). Over the past two decades, as sustainability has emerged as a global movement, policymakers and scholars have increasingly prioritized the environmental impacts of these events (5–10), positioning event greening as a crucial dimension of the sport industry. The International Olympic Committee (IOC), for example, has integrated environmental protection into its core sustainability agenda, which promoted many large-scale sport events to adopt comprehensive environmental protection plans across pre-, during, and post-event phases (11–13). Event greening is no longer a mere marketing slogan, rather, it has evolved into a lifestyle paradigm for individuals, a cost-efficiency strategy for event organizers, and a cornerstone of long-term community resilience (14, 15). Despite the growing importance of event greening, existing research predominately focuses on the event-level operations (16, 17) or venues-specific sustainability measures (18). A significant gap persists in understanding how these green legacies foster broad social acceptance and influence individuals' pro-environmental behaviors.

Environmental legacy, which refers to the long-lasting ecological and behavioral changes resulting from event-related sustainability initiatives, has garnered growing scholarly attention (4, 8, 19). For instance, Karadakis and Kaplanidou (19) found that both host and non-host residents ranked environmental legacy, surpassing economic and cultural legacies, as the most important aspect of event legacies for host communities across pre-, during, and post-stages. The findings align with Andereck et al. (20), who identified the environmental legacy as the strongest predictor of quality of life in their analysis. Prior research demonstrates that large-scale sport events foster environmental legacy by raising stakeholders' environmental awareness (21, 22). It is unclear how these legacies translate into residents' pro-environmental behaviors (PEBs). Wong et al. (10) argued that awareness alone rarely predicts sustained PEBs. Toscani et al.'s (4) systematic review of event environmental sustainability shows a methodological imbalance: existing studies pay more attention to event-level environmental impacts and green management practices, neglecting host community residents' perception of environmental legacy (PEL). This oversight is critical, as PEL plays a vital role in fulfilling large-scale sport events' core mission of delivering enduring benefits to host communities. However, the condition under which PEL influences residents' pro-environmental attitudes and then predicts meaningful PEBs remains under-researched.

The Norm Activation Model (23) posits that behavioral change requires personal responsibility and the belief that individual actions could drive meaningful outcomes. In the context of large-scale sport events, this study introduces two key constructs: perceived fit (PFT), which is defined as the cognitive alignment between environmental legacy and community's sustainable agenda, and authenticity evaluation (AUTH), which reflects residents' trust in PEL. Building on the NAM, the current study aims to examine how host community residents' PEL activates PEBs through the mediation of PFT. In addition, we assess whether AUTH moderates the relationship between PEL and PEBs. The 2023 Hangzhou Asian Games (2023 HAG), with their strong commitment to environmental sustainability, provides a unique context to examine these relationships. By addressing the research gaps, the current study not only deepens the theoretical understanding of how PEL activates PEBs via cognitive alignment but also clarifies AUTH's role in strengthening the relationship. Ultimately, these findings will provide actionable insights for policymakers and event organizers to design more effective environmental sustainability initiatives to foster long-term PEBs among residents, thereby contributing to sustainable urban development.

## 2 Theoretical framework

### 2.1 Large-scale sport event

Large-scale sport events are categorized as temporary, high-attendance occasions with substantial media reach, significant cost, and notable impacts on the environments and populations, such as the Olympic Games and FIFA World Cup. These events are widely recognized as catalysts for urban, social, and economic development in host communities (8, 24–26). Scholars in sport management and related fields have extensively examined the legacies of planning and hosting large-scale sport events, including urban regeneration (27), infrastructure upgrading (28), community pride (29) and quality of life enhancement (30, 31). The current study categorized the 2023 HAG as a large-scale sport event for the following reasons. First, the 2023 HAG attracted 11830 athletes and 5711 team officials from 45 Asian nations and territories, as well as 13 million visitors (32). Second, commercially, the 2023 HAG was highly successful by selling over 610 million tickets and generating 5.316 billion RMB in marketing revenue (33). Third, the event attracted 41.423 billion domestic viewers (34), while international mainstream media outlets such as ESPN, Olympic Channel, and Japan's NHK reached approximately 2.5 billion audiences. Furthermore, the event drove significant urban transformation through a 224.8 billion RMB investment, including 480 km of urban expressways, 800 km of highways, 516 km of metro lines, and 376 km of high-speed rail (32). Collectively, these attributes align with the defining criteria of a large-scale sport event, making the 2023 HAG an ideal context for this study.

### 2.2 Perceived environmental legacy

PEL is defined as residents' perceptions of the enduring environmental advancements associated with event hosting (6, 19, 35, 36). PEL encompasses residents' awareness of green improvements, such as upgraded land planning, expanded urban parks and green areas, increased sports and recreation areas, and heightened awareness of local natural resources conservation (15, 37). It also includes the underlying sense of responsibility fostered by an event's green initiatives, such as promoting green transportation, encouraging resource reuse, and advocating for sustainable material practices. However, compared with the object-based legacies (e.g., large-scale stadium), residents' awareness, perceptions and behavioral changes are often neglected in scholarship (38, 39). Existing studies often focus on physical outcomes, such as the management of large-scale stadiums in the post-event stage, while overlooking residents' PEL. This gap contradicts the literature's emphasis on environmental sustainability.

### 2.3 The Norm Activation Model

The NAM (23), originally developed as a general theory of altruism, is an internalized norm-based model designed to explain prosocial behaviors (e.g., helping, donating, volunteering), and PEBs, such as public transportation use and filing environmental complaints (23, 40–42). This socio-psychological model depicts the dynamic relationships among activators, personal norms, and prosocial behaviors. According to NAM, norm activation is a process through which individuals develop self-standards for prosocial conduct. Personal norms are activated when individuals recognize the negative consequences of inaction (i.e., awareness of consequences) and attribute responsibility for addressing those consequences to themselves (i.e., ascription of responsibility), thereby motivating prosocial behaviors. Prior studies in environmental psychology have empirically validated the applicability of NAM in predicting general PEBs (41, 43, 44), which are categorized as prosocial behaviors aimed at benefiting others or the environment without direct reciprocity. Accordingly, this study employs NAM as a theoretical foundation to examine the relationship between residents' PEL and PEBs in the context of large-scale sport events. Specifically, this study tests whether the activator-norm-behavioral psychological pathways posited by the NAM remains robust in explaining behavioral intentions associated with green events legacy.

## 3 Hypothesis development

**Figure 1:**
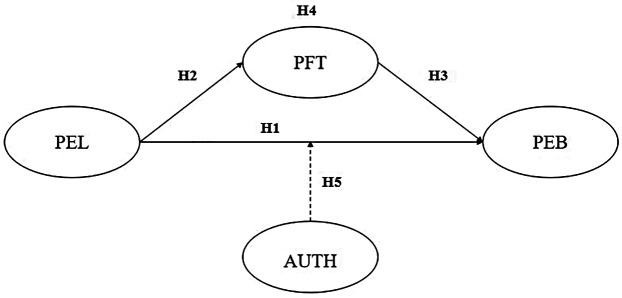
Hypothesized path model. PEL, perceived environmental legacy; PFT, perceived fit; AUTH, authenticity evaluation; PEB, pro-environmental behaviors.

To address the purpose of the current study, a conceptual framework was developed based (see Figure 1) on the NAM and related empirical studies (15, 41, 44–48). The framework specified hypothesized relationships between PEL, PFT and PEBs. The residents' AUTH was predicted to moderate the relationship between PEL and PEBs.

### 3.1 PEL influencing residents’ PEBs

Recent research highlights the growing salience of raising host community residents' awareness of environmental protection in shaping their subsequent PEBs (49, 50). PEBs, defined as prosocial intentions that consciously minimize harm to the environment or even benefit it (51), are increasingly recognized as indispensable for advancing sustainability goals. Scholars argue that perceptions of positive environmental outcomes plays a crucial role in motivating PEBs, often surpassing the influence of awareness of negative outcomes as it pertains to their quality of life (19, 52).

In the context of large-scale sport events, residents' active engagement in PEBs relates to their positive PEL. Prior studies suggest that when individuals recognize and value the environmental improvements generated by such events, they are more likely to adopt PEBs in their daily lives (53). This relationship is further supported by the NAM, which posits that awareness of positive consequences activates residents' sense of environmental responsibility, thereby motivating PEBs. Specifically, PEL fosters a moral obligation (e.g., “I should contribute to preserving these environmental gains”), which serves as a psychological mechanism linking environmental awareness to behavioral intention. For instance, residents with a stronger PEL demonstrate higher participation in electricity saving, public transportation use and other PEBs (40, 42, 45). Moreover, the clarity of PEL has been shown to strengthen residents' commitment to environmentally friendly behaviors, providing empirical support for the direct influence of PEL on behavioral outcomes. Therefore, the following hypothesis can be proposed:

• **H1:** PEL has a positive association with PEBs.

### 3.2 Mediating effect of PFT between PEL and PEBs

Simmons and Becker-Olsen (54) defined fit as the perceived degree of congruence between a cause and a brand (i.e., their perceived compatibility). The fit is often interchangeable with concepts such as congruence (55, 56), and similarity (57, 58). Congruence theory explains the value of fit by addressing how perceived similarity relates to attitudinal and relational outcomes. The theory posits that congruence between a brand and a cause enhances information processing by facilitating the storage and retrieval of associated memory (1, 59). In particular, higher levels of fit are linked to moral enhancement (45), as congruence pairings are processed as coherent and meaningful. Furthermore, strong fit fosters long-term relationship between the two parties by building trust and perceived alignment of values (60).

The objective of examining congruence is to assess the perceived compatibility between the 2023 HAG environmental legacy and the community's sustainable agenda. PFT is important because it can impact easily on how residents process information related to the 2023 HAG and its associations by increasing the clarity of the environmental legacies positioning, ultimately leading to positive attitudes toward the cause partner (54, 61). In our research context, we argue that when individuals perceive initiatives such as regenerated urban green spaces or sustainable sport facilities as meaningful environmental legacies (high PEL), this appraisal triggers three psychological mechanisms through PFT that could activate PEBs: (a) normative reinforcement, (b) value congruence, and (c) behavioral validation. Crucially, the fit between PEL and PEBs is contingent on the visibility and community relevance of the environmental legacy, which activate PEBs by enhancing perceived alignment with the community's sustainable agenda. This leads to the hypotheses:

• **H2:** PEL has a positive association with PFT.
• **H3:** PFT has a positive association with PEBs.
• **H4:** PFT mediates the relationship between PEL and PEBs.

### 3.3 Moderating effect of AUTH between PEL and PEBs

Authenticity encompasses concepts such as truthfulness, factual accuracy, honesty, genuineness, trustworthiness, and actuality (61). Traditionally, framed within object-based frameworks that distinguish between objective authenticity and constructive authenticity, the concept has evolved to emphasize experiential dimensions (62, 63). Early theories focused on the intrinsic attributes of objects or their symbolic meanings but were limited in capturing the subjective nature of human experiences. This limitation spurred the development of existential authenticity, a framework that shifts focus from object-oriented criteria to individuals' personal experiences of genuineness and self-realization. In the context of large-scale sport event environmental legacy, this experiential perspective becomes particularly relevant, as it extends beyond physical infrastructure to examine how residents emotionally engage with and interpret sustainability initiatives. The existential approach explains why communities may embrace PEL as authentic while rejecting those initiatives viewed as inauthentic or performative (i.e., greenwashing), offering a nuanced understanding of resident responses to environmental interventions.

Based on NAM, we posit that residents’ AUTH as a credibility amplifier, strengthening the positive effect of PEL on PEBs by resolving distrust, clarifying impact visibility, and reinforcing cultural relevance. For PEL to effectively motivate PEBs, it must be perceived as authentic which aligns with residents’ expectation of truthfulness and serves as a validating force for their environmental commitments. This AUTH not only enhances PEL's salience but also moderates the relationship between PEL initiatives and residents' behavioral intentions. Thus, we formulated the following hypothesis:

• **H5:** AUTH moderates the relationship between PEL and PEBs.

## 4 Method

### 4.1 Setting

The 2023 HAG was selected as the research context due to its unprecedented commitment to environmental sustainability. As the first Asian Games to fully incorporate the United Nations Sustainable Development Goals into its planning framework, and to be presented the Olympic Council of Asia Sports and Environmental Award, it established a benchmark for sustainable large-scale sport events, leaving behind a comprehensive environmental legacy that continues to shape the city's ecological landscape and residents' daily practices.

This environmental legacy manifests in both tangible and intangible forms: from the solar-powered “Big Lotus” stadium that now serves as a community landmark, to the expanded green transportation network integrating 50 kilometers of new cycling lanes with the metro system, and the ecological restoration projects along the Qiantang River that have enhanced urban biodiversity. The intangible environmental legacy is equally significant, particularly the enduring promotion of green concepts. As the first carbon-neutral Asian Games, Hangzhou was the first to employ digital technologies to convey environmental messages-replacing traditional fireworks with digital pyrotechnics, utilizing 3D animations, and featuring virtual “torchbearers.” These initiatives effectively communicated sustainability principles to global audiences. Moreover, the Games' green philosophy has been consistently maintained in post-event operations, demonstrating a long-term commitment to environmental stewardship that extends far beyond the closing ceremony.

### 4.2 Participants and data collection

A cross-sectional online survey was administered through Wenjuanxing (i.e., a licensed online panel) to Hangzhou residents from December 10 to December 17, 2024. A stratified convenient sampling approach was applied based on Hangzhou's administrative districts to ensure geographic diversity. A total of 823 valid responses were collected, achieving a response rate of 82.5%. The sample comprised 52% female and 48% male respondents, with an average age of 34.7 years (SD = 9.2). Education levels varied, with 68% holding a bachelor's degree or higher. Additionally, regarding occupational background, 45% were white-collar employees, 28% were blue-collar workers, and 27% were in other sectors. Monthly household income showed 22% earning below USD$700, 49% were between USD$701 and USD$1,500, and 29% were above USD$1,501. The overall demographics reflect Hangzhou's highly educated population.

### 4.3 Measures

All constructs were measured using a 7-point Likert scale (1 =“strongly disagree,” 7 = “strongly agree”), adapted from established scales and validated for this study. Back-translation ensured linguistic accuracy.

PEL was measured using items adapted from a 3-item scale (64) that assessed residents' perceptions of the Games' environmental legacy. PFT was assessed by a 6-item scale (1) that evaluated the alignment between event initiatives and community's sustainable agenda. AUTH was assessed by using an 8-item scale (65) that gauged residents' evaluation of green authenticity. In addition, PEBs was assessed by a 4-item scale (66) that captured behavioral intentions. Full item wordings are listed in Appendix 1.

Given that the items were originally in English and the questionnaire targets Chinese speakers, we adopted the back-translation protocol by Doherty, Chen, and Alexander (67). One bilingual researcher translated English to Chinese, and then another one back-translated Chinese into English. Finally, an English native speaker cross-validated the two English versions to resolve discrepancies, ensuring the Chinese questionnaire matches the original intent and safeguards validity.

### 4.4 Data analysis

Structural equation modeling (SEM) was employed to test the hypotheses using the Mplus 8.32 statistical package. Overall model fit was evaluated using goodness-of-fit indices. According to Hu and Bentler (68), cutoff values exceeding 0.95 for the comparative fit index (CFI), less than 0.08 for the standardized root mean square residual (SRMR) and below 0.06 for the root mean square error of approximation (RMSEA) were used to indicate acceptable model fit. Factor loading exceeding 0.60 (69), average variance extracted (AVE) values above 0.50, construct reliability (CR) values greater than 0.70, and McDonald's *ω* values greater than 0.70 (70) supported the reliability and validity of the measured constructs.

Mediation analysis was performed using a non-parametric bootstrapping procedure with 5,000 resampling iterations to compute bias-corrected 95% confidence intervals (CI) for indirect effects. An indirect effect was considered significant if its 95% CI excluded zero (67).

The moderating effect of AUTH on the relationship between PEL and PEBs was examined using latent moderated structural modeling (LMS) (71). A latent interaction variable (PEL X AUTH) was constructed and included in the structural model as a predictor of PEBs. As goodness-of-fit indices are unavailable in LMS, model fit was assessed by comparing two nested models via a log-likelihood ratio test (71). Model 0 (the baseline model) comprised all direct paths and served as the reference, while Model 1 (the hypothesized model) incorporated the interaction variable to test the moderating effect.

## 5 Results

### 5.1 Testing of data normality

A Skewness-Kurtosis tests were conducted to assess the data normality prior to SEM (71). Results indicated Skewness values ranging from −1.73 to 0.53, and Kurtosis values falling between −0.53 and 7.49, suggesting no significant deviations from normality that would bias the SEM results.

### 5.2 Testing of multicollinearity

Prior to conducting the main analyses, multicollinearity among the four focal factors (PEL, PFT, PEBs, and AUTH) was assessed employing variance inflation factor (VIF) and tolerance as diagnostic metrics. The results indicated no evidence of severe multicollinearity: PEL yielded a VIF of 1.23 (tolerance = 0.81), PFT had a VIF of 1.18 (tolerance = 0.85), PEBs showed a VIF of 1.31 (tolerance = 0.76), and AUTH exhibited a VIF of 1.27 (tolerance = 0.79). All VIF values were well below the commonly accepted threshold of 10 (70), and all tolerance values exceeded the critical cutoff of 0.1 (70). These findings confirm that multicollinearity does not pose a threat to the current study, ensuring the reliability of coefficient estimates in subsequent statistical analyses.

### 5.3 Common method variance

Given the potential risk of common method variance (CMV) inherent in cross-sectional surveys where predictors and outcomes are self-reported by a single respondent, this study adopted a combination of proactive preventive measures and *post-hoc* statistical tests to mitigate and assess CMV. Prior to data collection, we implemented procedural safeguards: separating the measurement of independent variables (e.g., PEL, PFT) and dependent variables (e.g., PEB) across different sections of the questionnaire, assuring respondents of full anonymity to reduce social desirability bias, and using clear, non-leading wording for all items.

After data collection, we conducted Harman's single-factor test via unrotated exploratory factor analysis on all scale items. The results showed that the first unrotated factor explained 28.7% of the total variance, well below the 40% threshold indicating severe CMV. Additionally, a common latent factor (CLF) test revealed no significant improvement in model fit when a latent method factor was added (Δ*χ*^2^/Δdf = 1.23, *p* > 0.05). Together, these results confirm that common method bias does not pose a substantial threat to the validity of the study's findings.

### 5.4 Testing of the measurement model

The goodness-of-fit indices of the measurement model demonstrate a good data-model fit [$\chi^{2}$(389.23)/df(164) = 2.37; CFI = 0.98; RMSEA = 0.04; SRMR = 0.03]. As shown in Table 1, standard factor loadings of all the latent variables are ranged from 0.64 to 0.91, exceeding the recommended threshold of 0.60 (69). The latent variables have AVE values between 0.53, 0.68, CR values ranged from 0.70 to 0.91, and McDonald's *ω* exceeded 0.83, confirming the construct reliability and convergent validity of the multiple item scales. For discriminant validity, Table 2 indicates that the square roots of AVE values for PEL (0.73), PFT (0.81), PEBs (0.82) and AUTH (0.77) exceed the inter-construct correlation coefficients satisfying the Fornell-Larcker criterion. As further support of discriminant validity, the HTMT ratios across all constructs are smaller than 0.85 (69).

**Table 1:** Psychometric properties of all items analyzed in the measurement model.

Construct/Items	Loading	CR	*ω*	AVE
Perceived Environmental Legacy (PEL)		0.77	0.83	0.53
Asian Games seem to be environmentally responsible.	0.77			
Asian Games look like a good event that cares the environment.	0.70			
Asian Games do a lot in environmental protection for the community.	0.71			
Perceived Fit (PFT)		0.93	0.95	0.64
The environmental performance is logically related to the Asian Game	0.81			
The environmental performance is relevant to residents of Hangzhou	0.80			
It is very plausible that Asian Games engage in environmental performance like this	0.89			
It is typical of Asian Games to engage in environmental performance like this	0.84			
The environmental performance is consistent with the image of Asian Games	0.82			
Overall, the environmental performance is a good match with the Asian Games	0.86			
Pro-environmental Behaviors (PEBs)		0.89	0.92	0.68
I intend to reduce my environmental footprint as much as possible	0.91			
I intend to do all I can to help reduce climate change	0.82			
I intend to treat the environment as respectfully as possible	0.71			
I intend to act as sustainably as I can	0.81			
Authenticity Evaluation (AUTH)		0.89	0.94	0.59
The environmental initiatives of the Asian Games are truthful and genuine	0.73			
The environmental legacy of the Asian Games is consistent with my understanding of what the Games stand for	0.78			
The environmental efforts of the Asian Games reflect its unique identity in a believable way	0.83			
The Asian Games’ environmental actions are aligned with its stated values and mission	0.70			
The Asian Games stayed true to itself through its environmental actions	0.83			
The Asian Games are stood up for what it believes in	0.76			

*N* = 823; all standardized factor loadings were significant (*p* < 0.01); CR, composite reliability coefficients; AVE, average variance extracted.

**Table 2:** Descriptive statistics and discriminant validity tests of the measurement model.

Constructs	PEL	PFT	PEBs	AUTH
PEL	0.73			
PFT	0.61**	0.81		
PEBs	0.11	0.31**	0.82	
AUTH	0.21*	0.09	0.16*	0.77
HTMT				
PEL	1			
PFT	0.80	1		
PEBs	0.35	0.53	1	
AUTH	0.21	0.17	0.20	1
Mean	6.01	5.83	5.99	5.23
S.D.	0.83	1.21	1.07	1.54

*N* = 823. The top matrix shows the results of the discriminant validity test applying Fornell and Larcker's (79) criterion; in this matrix, diagonal values represent the square-rooted average variance extracted, and the remaining values represent correlations between the constructs. The bottom matrix shows the results of the discriminant validity test applying HTMT_85_ criterion. M, mean; SD, standard deviation.

* *p* < 0.05.

** *p* < 0.01.

### 5.5 Determining factor constructs

Prior to testing the structural model, a series of competing model comparisons were conducted to validate the factor structure of the latent variables and enhance the rigor of factor structure determination. Three sets of models were constructed for comparison: Model 1 was the four-factor baseline model (i.e., PEL, PFT, AUTH, PEB) to be validated in this study; Model 2 was the three-factor model obtained by merging PFT and PEL (i.e., PEL + PFT, AUTH, PEB); and Model 3 was the single-factor model. Table 3 showed that Model 1 exhibited the best fit. Further chi-square difference test between Model 1 and Model 2 revealed that the four-factor model fit significantly better than the three-factor model [*Δχ*^2^(3) = 1,170.307, *p* < .001]. This result provides strong statistical support for the discriminant validity among the four latent variables, indicating that they represent distinct constructs.

**Table 3:** Summary of model comparison.

Construct	$\chi^{2}/df$	CFI	RMSEA	SRMR
Four-factor model	2.29	0.98	0.04	0.03
Three-factor model	12.32	0.78	0.12	0.15
Single-factor model	23.97	0.55	0.16	0.14

### 5.6 Testing of the structural model

The goodness-of-fit indices of the structural model demonstrated appropriate data-model fit: [$\chi^{2}$(117.04)/df(51) = 2.29; CFI = 0.98; RMSEA = 0.04; SRMR = 0.03]. Table 4 summarizes the standardized path coefficients, t-values, *p*-values, and the bias-corrected 95% CI of the hypothesized model. Specifically, the direct association between PEL and PEBs was insignificant (*β* = 0.06, *p* = 0.15), leading to the rejection of H1. To test the mediating effect of PFT, we first evaluated the direct paths from PEL to PFT and PFT to PEBs. As shown in Table 4, the association between PEL and PFT is significant and positive (*β* = 0.61, *p* < 0.01) which supports H2. The association between PFT and PEBs was also significant and positive (*β* = 0.29, *p* < 0.01), confirming H3. Bootstrapping results in Table 5 revealed a significant indirect effect of PEL on PEBs via PFT. The indirect effect of PFT is significant within the relationship between PEL and PEBs as its 95% CI excludes zero [*β* = 0.18, *p* < 0.01, 95% CI = (0.11, 0.28)]. Thus, H4 is supported. Given the insignificant direct effect of PEL and PEBs in the presence of the mediator, PFT fully mediates the relationship between PEL and PEBs.

**Table 4:** Summary for path coefficients of hypothesized paths.

	Hypothesized paths	Model 0	Model 1
H1	PEL → PEBs	0.09	0.11*
H2	PEL → PFT	0.61**	0.36**
H3	PFT → PEBs	0.29**	0.19*
	AUTH→ PEBs	–	0.15*
H5	AUTH×PEL→ PEBs	–	0.18

*N* *=* *823.* Model 0 is a linear model without the moderator (AUTH); Model 1 is the hypothesized model including the moderator.

* *p* < 0.05.

** *p* < 0.01.

**Table 5:** Bootstrapping results of hypothesized indirect effects.

Hypothesized paths	β	S.E.	Bootstrapping (95% CI)	
			Lower 2.5%	Upper 2.5%
H4: PEL → PFT → PEBs	0.18**	0.05	0.10	0.28

*N* *=* *823.* Β*,* standardized coefficient; S.E., standard error.

** *p* < 0.01.

### 5.7 Testing of the moderating effect

**Figure 2:**
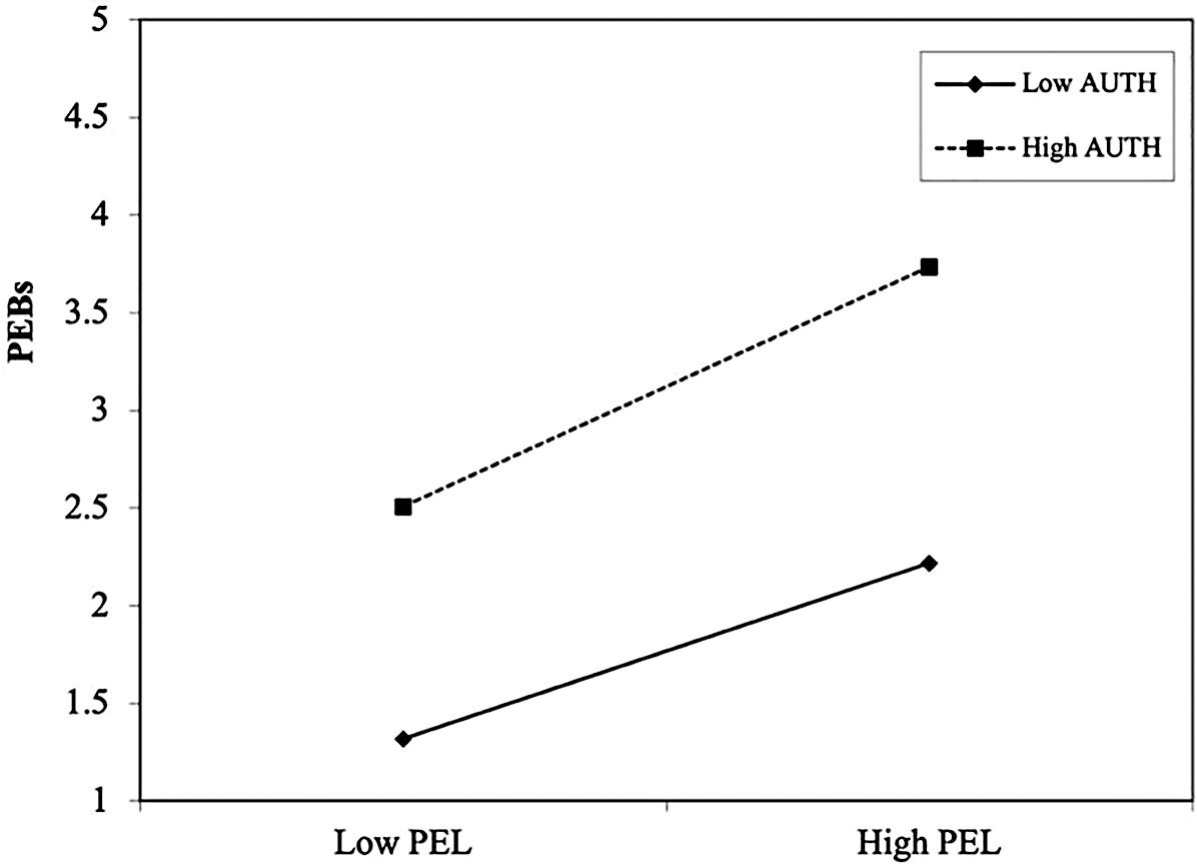
AUTH moderation plot.

The log-likelihood ratio test ($\Delta \chi^{2}(2)=5.72,\;p<0.01$) comparing Model 0 and Model 1 indicated that Model 1 yielded a better fit than Model 0. It confirms the interaction term (AUTH × PEL) captures unique variance beyond the baseline model and supports AUTH as a moderator. As shown in Table 4, the standardized interaction effect of AUTH × PEL on PEBs was statistically significant (*β* = 0.18, *p* < 0.05), hence supporting H5. This means a 1 SD increase in AUTH strengthened the positive association between PEL and PEBs by 0.18 SD (see Figure 2). These findings confirm AUTH acts as a facilitative moderator, enhancing the association between PEL and PEBs when residents' PEL is authentic.

## 6 Discussion

This research examined the relationship between host community residents' PEL and PEBs through the mediation of PFT. We also assessed AUTH as a moderator of this relationship. Results indicated that PEL exerts a positive relation on PEBs with PFT acting as a full mediator. This finding corroborates existing studies suggesting that PFT, as a cognitive construct reflecting residents' perceptions of congruence between PEL and community's sustainable agenda, facilitates PEBs. Furthermore, the findings align with theoretical applications of the NAM in sport event and environmental psychology research, illustrating the psychological pathway from environmental awareness to behavioral intention. This further validates and expands the utility of NAM in the context of large-scale sport events by demonstrating how cognitive processes activate host community residents' PEBs. Notably, the current study revealed a significant moderating effect of AUTH within the framework. This moderation indicated when residents' PEL is authentic, they exhibit greater willingness to engage in PEBs.

The insignificant direct relationship between PEL and PEBs can be explained by the following reasons. As environmental concerns grow in prominence (2, 66), event organizers increasingly adopt green practices to garner community support. This strategic alignment with environmental goals yields dual benefits: it fulfills social responsibility while enhancing marketing effectiveness through improved resident attitudes and enhanced resident environmental responsibility (72, 73). The critical mechanism underpinning this relationship is PFT which reflects the congruence between an event's environmental legacies and a community's sustainable agenda. This explains why the effect of PEL on PEBs is fully mediated by PFT. Without perceived value alignment, PEL fails to activate residents' sustainable behavioral intention. The congruence perspective thus reveals how strategic environmental legacy programs can simultaneously achieve ecological and competitive advantages (60).

### 6.1 Theoretical implication

There are several theoretical contributions that need to be highlighted. First, this study applied the NAM to explain how residents' PEBs are associated with their PEL which contributes to a comprehensive understanding of the impact of the environmental legacy of large-scale sport events. Within the current research context, NAM offers a theoretical lens to analyze the psychological drivers of residents' PEBs. The framework posits that personal norms are pivotal in triggering behavioral change, with PEL serving as a catalyst for norm activation. Compared with previous research using Theory of Planned Behavior (TPB) and Social Exchange Theory (SET), NAM offers distinct advantages in explaining the psychological mechanisms of PEBs. TPB emphasizes that behavior is shaped by attitudes, subjective norms, and perceived behavioral control. While TPB and SET offer valuable insights (53, 74), they may not fully capture the moral-obligation-based motivation central to environmental behaviors in this context. For instance, Regulatory Fit Theory (59) could further explain why PFT is so powerful: when the event's legacy aligns with the community's sustainable agenda, it creates a feeling of ‘rightness’ that enhances motivational strength. Furthermore, identity-based theories help explain the role of AUTH; if residents incorporate the event's environmental values into their self-concept, their behaviors are more likely to be consistent and enduring. Thus, our integration of PFT and AUTH within the NAM framework bridges cognitive, normative, and identity-based pathways to behavior.

Second, this study confirms that PFT fully mediates the relationship between PEL and PEBs. This full mediation reveals that the traditional NAM factors are insufficient to explain the relationship. Integrating PFT into the NAM framework bridges the gap between environmental perception and behavioral outcomes. PFT strengthens the connection by enhancing residents' sense of environmental responsibility. When residents perceive a high level of congruence between PEL and their community's sustainable agenda, their sense of environmental responsibility intensifies. This process amplifies the activation of personal norms and thus promotes PEBs. These findings validate the NAM's core premise while expanding its application to the unique context of sport event environmental legacy research.

Third, by confirming the significant moderating effect of AUTH, this study enriches the theoretical understanding of the relationships among PEL, PFT, and PEBs. Given AUTH was operationalized via 5-point scale items (e.g., authenticity of content cues) with a sample SD of 0.79, a meaningful improvement (e.g., 0.5 SD, 0.4 points on the scale) in AUTH would enhance the PEL-PEBs link by 0.09 SD. This moderation suggests that when residents' PEL is authentic, they are more likely to engage in PEBs. This highlights the critical role of authenticity in fostering environmental behavioral intention. By disentangling these psychological mechanisms, the current study advances a refined theoretical model that clarifies how PEL is associated with residents' PEBs through cognitive alignment and normative validation, with authenticity serving as a contextual amplifier.

### 6.2 Managerial implication

This study offers several actionable insights for policymakers and event organizers. The effect sizes offer tangible insights for intervention. For example, the strong effect of PEL on PFT suggests that effectively communicating the alignment between event legacies and community goals is paramount. The moderating effect of AUTH indicates that improving the perceived authenticity of environmental initiatives can meaningfully strengthen the direct impact of legacy perceptions on residents’ behavioral intentions. These insights align with frameworks with guidance for maximizing event values. Furthermore, event organizers need to adopt robust environmental protection policies to minimize ecological harm during event planning and execution. These findings align with Chalip's (75) social leveraging model, while expanding it to include contextual factors specific to large-scale sport events, such as accessibility and equity considerations. Operationally, our study demonstrates that event organizers and relevant stakeholders can achieve well-being outcomes by embedding event venues in local communities through ensuring ancillary events (e.g., live sites) are accessible and participatory for all.

Marketing complements operational decision-making by ensuring community awareness and elevating the host community's international profile. Event organizers and relevant stakeholders can maximize environmental legacy by implementing marketing strategies that provide updates on the environmental progress, share information about event assets to build familiarity, promote event commitments to equity and inclusion, and encourage participation in ancillary events. Event organizers and planners should further communicate accurate impacts that consider both the potential positive and negative impacts that residents may experience since authenticity evaluation is a key construct to activate PEBs. As Fredline (76) suggests, when residents are transparently informed of an event's benefits and perceive these impacts firsthand, the event is more likely to be viewed as successful, fostering support for future initiatives. For instance, if major environmental initiatives are undertaken by the host city, then pertinent media communications have to take place to allow the proper assessment of these legacies by the residents. This study offers several actionable insights for policymakers and event organizers. To effectively activate host community residents' PEBs, policymakers and event organizers should prioritize aligning residents' PEL with the community's sustainable agenda as these are what they could perceive and preserve. This involves ensuring that legacies such as urban green spaces and sustainable facilities not only are physically implemented but also resonate with what the community and residents value. Additionally, maintaining high levels of AUTH in environmental legacy is crucial, as this fosters residents' trust and strengthens their motivation to engage in PEBs.

### 6.3 Limitation and future direction

It is important to acknowledge the limitations of this study and related future research opportunities. Foremost, in the theoretical framework, there may be other variables in addition to PFT and AUTH, and further study should extract other key psychological variables, such as commitment (77), loyalty (78), and involvement (72) into the theoretical framework to enhance its comprehensiveness. Second, our data were collected one-year post-event and from a single city, which captures initial perceptions but cannot speak to the long-term evolution of PEL or its generalizability to other cultural contexts. The cross-sectional nature of our data necessitates caution in interpreting causality. While our model is grounded in the theoretically driven NAM framework, the observed relationships are associative. For instance, it is plausible that residents with stronger pre-existing pro-environmental tendencies are more likely to perceive and value the event's environmental legacy. Future longitudinal or experimental designs are needed to robustly establish the causal direction proposed in our model. Third, the sample's overrepresentation of highly educated individuals may restrict generalizability to populations with lower educational attainment, warranting caution when extending findings broadly. Future research could expand sampling frames to include more respondents with lower educational backgrounds, such as vocational school graduates, to enhance external validity. Finally, the high mean scores on PEL and PEBs suggest a potential ceiling effect, which may limit the variability and attenuate the observed relationships. Diverse analytical methods such as neural networks and Hierarchical Linear Modeling should be used to test the theoretical model.

## 7 Conclusions

This research demonstrates that the PEL of large-scale sport events activates residents' PEBs through a psychological path mechanism: PFT fully mediates the relationship by aligning PEL with the community's sustainable agenda, as well as AUTH moderates the direct relationships between PEL and PEBs. These findings redefine environmental legacy success metrics beyond physical infrastructure to include psychological and behavioral impacts. For cities hosting future sustainable sports events, our study provides an evidence-based framework to maximize lasting environmental benefits for those who prioritize resident-oriented design, transparent implementation, trustworthy legacy and continuous engagement. As the sport event industry evolves toward greater sustainability, understanding these human dimensions of environmental legacy will be crucial for achieving meaningful, long-term environmental behavioral change.

## Data Availability

The original contributions presented in the study are included in the article/Supplementary Material, further inquiries can be directed to the corresponding author.

## Appendix 1

**Table:**

Construct/Items
Perceived Environmental Legacy (PEL)
The Asian Games seem to be environmentally responsible
The Asian Games look like a good event that cares about the environment
The Asian Games do a lot for environmental protection for the community
Perceived Fit (PFT)
The environmental performance is logically related to the Asian Games
The environmental performance is relevant to residents of Hangzhou
It is very plausible that the Asian Games engage in environmental performance like this
It is typical of Asian Games to engage in environmental performance like this
The environmental performance is consistent with the image of the Asian Games
Overall, the environmental performance is a good match with the Asian Games
Pro-environmental Behaviors (PEBs)
I intend to reduce my environmental footprint as much as possible
I intend to do all I can to help reduce climate change
I intend to treat the environment as respectfully as possible
I intend to act as sustainably as I can
Authenticity Evaluation (AUTH)
The Asian Games’ environmental performances are genuine
The environmental performances preserve what the Asian Games means to me
The environmental performances capture what makes the Asian Games unique to me
The Asian Games’ environmental performances are in accordance with the Asian Games’ values and beliefs
The Asian Games are being true to itself with its environmental performances
The Asian Games stand up for what it believes in

## References

[1] Habitzreuter AM Koenigstorfer J. The impact of environmental CSR-linked sport sponsorship on attitude toward the sponsor depending on regulatory fit. J Bus Res. (2021) 124:720–30. 10.1016/j.jbusres.2018.11.040

[2] McCullough B. Pressures from stakeholders to implement environmental sustainability efforts: an overview. In: Casper JM Pfahl ME, editors. Sport Management and the Natural Environment: Theory and Practice. London: Routledge (2015). p. 88–98.

[3] Shin N Welty Peachey J. Understanding the global-local nexus in the context of the Olympic games: implications for managing community development through sport mega events. J Sport Manag*.* (2022) 36:82–95. 10.1123/jsm.2020-0380

[4] Toscani AC Vendraminelli L Vinelli A. Environmental sustainability in the event industry: a systematic review and a research agenda. J Sustain Tour. (2024) 32:2663–97. 10.1080/09669582.2024.2309544

[5] Alonso-Vazquez M Packer J Fairley S Hughes K. The role of place attachment and festival attachment in influencing attendees’ environmentally responsible behaviors at music festivals. Tour Recreat Res. (2019) 44:91–102. 10.1080/02508281.2018.1545393

[6] Kearney AT. Building a Legacy- Sports Large-Scale-Events Should Last a Lifetime. Chicago, IL: AT Kearney Inc (2007).

[7] Leopkey B Parent MM. Stakeholder perspectives regarding the governance of legacy at the Olympic games. Ann Leis Res*.* (2015) 18:528–48. 10.1080/11745398.2015.1092388

[8] Müller M. What makes an event a large-scale-event? Definitions and sizes. Leis Stud. (2015) 34:627–42. 10.1080/02614367.2014.993333

[9] Prayag G Hosany S Nunkoo R Alders T. London residents’ support for the 2012 Olympic games: the mediating effect of overall attitude. Tour Manag. (2013) 36:629–40. 10.1016/j.tourman.2012.08.003

[10] Wong IA Wan YKP Huang GI Qi S. Green event directed pro-environmental behavior: an application of goal system theory. J Sustain Tour. (2021) 29:1948–69. 10.1080/09669582.2020.1770770

[11] Alberts HC. The reuse of sports facilities after the winter Olympic games. Focus Geogr. (2021) 54:24–32. 10.1111/j.1949-8535.2010.00022.x

[12] Azzali S. The legacies of Sochi 2014 winter Olympics: an evaluation of the Adler Olympic park. Urban Res Pract. (2016) 10:329–49. 10.1080/17535069.2016.1216586

[13] Cantelon H Letters M. The making of the IOC environmental policy as the third dimension of the Olympic movement. Int Rev Sociol Sport. (2000) 35:294–308. 10.1177/101269000035003004

[14] Martinho G Gomes A Ramos M Santos P Gocalves G Fonseca M Pires A. Solid waste prevention and management at green festivals: a case study of the Andancas festival, Portugal. Waste Manag*.* (2018) 71:10–8. 10.1016/j.wasman.2017.10.020 29102358

[15] Yeerkenbieke G Chen C He G. Public perceived effects of 2022 winter Olympics on host city sustainability. Sustainability. (2021) 13:3787. 10.3390/su13073787

[16] Han H. The norm activation model and theory-broadening: individuals’ decision-making on environmentally- responsible convention attendance. J Environ Psychol. (2014) 40:462–71. 10.1016/j.jenvp.2014.10.006

[17] Park E Boo S. An assessment of convention tourism’s potential contribution to environmentally sustainable growth. J Sustain Tour. (2010) 18:95–113. 10.1080/09669580903147936

[18] Whitfield J Dioko LAN. Measuring and examining the relevance of discretionary corporate social responsibility in tourism: some preliminary evidence from the U.K. J Travel Res. (2012) 51:289–302. 10.1177/0047287511418369

[19] Karadakis K Kaplanidou K. Legacy perceptions among host and non-host Olympic games residents: a longitudinal study of the 2010 Vancouver Olympic games. Eur Sport Manag Q. (2012) 12:243–64. 10.1080/16184742.2012.680067

[20] Andereck KL Valentine KM Vogt CA Knopf RC. A cross-cultural analysis of tourism and quality of life perceptions. J Sustain Tour. (2007) 15: 483–502. 10.2167/jost612.0

[21] Mair J Laing JH. Encouraging pro-environmental behavior: the role of sustainability-focused events. J Sustain Tour. (2013) 21:1113–28. 10.1080/09669582.2012.756494

[22] Mair J Jago L. The development of a conceptual model of greening in the business events tourism sector. J Sustain Tour. (2009) 18:77–94. 10.1080/09669580903291007

[23] Schwartz SH. Normative influences on altruism. Adv Exp Soc Psychol. (1977) 10:221–79. 10.1016/S0065-2601(08)60358-5

[24] Kaplanidou K. Sport events and community development: resident considerations and community goals. Int J Sports Mark Spons. (2021) 22:53–66. 10.1108/IJSMS-05-2020-0082

[25] McGillivray D Edwards MB Brittain I Bocarro J Koenigstorfer J. A conceptual model and research agenda for bidding, planning and delivering Major sport events that lever human rights. Leis Stud. (2019) 38:175–90. 10.1080/02614367.2018.1556724

[26] Scandizzo PL Pierleoni MR. Assessing the Olympic games: the economic impact and beyond. J Econ Surv. (2018) 32:649–82. 10.1111/joes.12213

[27] Sánchez F Broudehoux AM. Large-Scale-Events and urban regeneration in Rio de Janeiro: planning in a state of emergency. Int J Urban Sustain Dev. (2013) 5: 132–53. 10.1080/19463138.2013.839450

[28] Swart K Bob U. Mega sport event legacies and the 2010 FIFA world cup. Afr J Phys Health Educ Recreat Dance. (2012) 18:1–11. 10520/EJC128313

[29] Kim W Jun HM Walker M Drane D. Evaluating the perceived social impacts of hosting large-scale sport tourism events: scale development and validation. Tour Manag. (2015) 48:21–32. 10.1016/j.tourman.2014.10.015

[30] Liu D. Social impact of major sports events perceived by host community. Int J Sports Mark. Spons. (2016) 17:78–91. 10.1108/IJSMS-02-2016-005

[31] Preuss H. The conceptualization and measurement of large-scale sport event legacies. J Sport Tour. (2007) 12:207–28. 10.1080/14775080701736957

[32] HAGOC. Hangzhou Asian Games Presents Final Report to OCA General Assembly (2024). Available online at: https://oca.asia/news/5464-hangzhou- asian-games-presents-final-report-to-oca-general-assembly.html (Accessed April 15, 2024).

[33] Hangzhou Net. Report on the Hosting of the Hangzhou Asian Games (Asian Para Games) and the Sustained Amplification of Their Legacy (2023). Available online at: https://z.hangzhou.com.cn/2023/rddswchy/content/content_8666436.html (Accessed April 15, 2024).

[34] China Media Group. 41.4 Billion Views! CMG’s Hangzhou Asian Games Broadcast Sets Record as Largest-Scale and Most-Watched Coverage in History (2024). Available online at: https://sinoreporter.com/headlines/157195/ (Accessed April 15, 2024).

[35] Chen S Xing X Chalip L. Planning and implementation of event leveraging strategy: China’s legacy pledge to motivate 300 million people to be involved in winter sport. Sport Manag Rev. (2022) 25:771–90. 10.1080/14413523.2021.1987737

[36] Tsaur SH Yen CH Tu JH Wang CH Liang YW. Evaluation of the 2010 Taipei international Flora exposition from the perceptions of host-city residents: a new framework for large-scale-event legacies measurement. Leis Stud. (2015) 36:65–88. 10.1080/02614367.2015.1037786

[37] Mirzayeva G Turkay O Akbulaev N Ahmadov F. The impact of mega-events on urban sustainable development. Entrep Sustain Issues. (2020) 7:1653–66. 10.9770/jesi.2020.7.3(15)

[38] Bob U Swart K. Resident perceptions of the 2010 FIFA soccer world cup stadia development in Cape Town. Urban Forum. (2009) 20:47–59. 10.1007/s12132-009-9052-2

[39] Konstantaki M Wickens E. Residents’ perceptions of environmental and security issues at the 2012 London Olympic games. J Sport Tour. (2010) 15:337–57. 10.1080/14775085.2010.533921

[40] Bamberg S Hunecke M Blöbaum A. Social context, personal norms and the use of public transportation: two field studies. J Environ Psychol. (2007) 27:190–203. 10.1016/j.jenvp.2007.04.001

[41] Steg L De Groot J. Explaining prosocial intentions: testing causal relationships in the norm activation model. Br J Soc Psychol. (2010) 49:725–43. 10.1348/014466609X477745 20021707

[42] Zhang Y Wang Z Zhou G. Antecedents of employee electricity saving behavior in organizations: an empirical study based on norm activation model. Energy Policy. (2013) 62:1120–7. 10.1016/j.enpol.2013.07.036

[43] Assaker G. Sustainability concerns on pro-sustainable travel behavior: combining the United Nations SDGs, norm activation model and value theory. J Hosp Tour Insights. (2025) 8:20–40. 10.1108/JHTI-01-2024-0012

[44] Whitmarsh L O’Neill S. Green identity, green living? The role of pro-environmental self-identity in determining consistency across diverse pro-environmental behaviors. J Environ Psychol. (2010) 30:305–14. 10.1016/j.jenvp.2010.01.003

[45] Godin G Conner M Sheeran P. Bridging the intention-behaviour “gap”: the role of moral norm. Br J Soc Psychol. (2005) 44:497–512. 10.1348/014466604X17452 16368016

[46] Landon AC Woosnam KM Boley BB. Modeling the psychological antecedents to Tourists’ pro-sustainable behaviors: an application of the value-belief-norm model. J Sustain Tour. (2018) 26:957–72. 10.1080/09669582.2017.1423320

[47] Sajid M Zakkariya KA Peethambaran M George A. Determinants of on-demand ridesharing: the role of awareness of environmental consequences. Manag Environ Qual. (2022) 33:847–63. 10.1108/MEQ-10-2021-0235

[48] Van Riper CJ Kyle GT. Understanding the internal processes of behavioral engagement in a national park: a latent variable path analysis of the value-belief-norm theory. J Environ Psychol. (2014) 38:288–97. 10.1016/j.jenvp.2014.03.002

[49] Bai J Tian Q Fan D Sun H. Perceived corporate social responsibility and employee voluntary pro-environmental behavior: does moral motive matter? Corp Soc Responsib Environ Manag. (2023) 31:816–30. 10.1002/csr.2603

[50] Latif B Ong TS Meero A Abdul Rahman AA Ali M. Employee-perceived corporate social responsibility (CSR) and employee pro-environmental behavior (PEB): the moderating role of CSR skepticism and CSR authenticity. Sustainability. (2022) 14:1380. 10.3390/su14031380

[51] Steg L Vlek C. Encouraging pro-environmental behaviour: an integrative review and research agenda. J Environ Psychol. (2009) 29:309–17. 10.1016/j.jenvp.2008.10.004

[52] Gursoy D Kendall KW. Hosting large-scale events: modelling locals’ support. Ann Tour Res. *(*2006) 33:603–23. 10.1016/j.annals.2006.01.005

[53] Moon K-K Lee S-H Jeong S-Y. Examining the relationship between individualism and pro- environmental behavior: the moderating role of social cohesion. Behav Sci. (2023) 13:661. 10.3390/bs13080661 37622801 PMC10451664

[54] Simmons CL Becker-Olsen KL. Achieving marketing objectives through social sponsorships. J Mark. (2006) 70:154–69. 10.1509/jmkg.70.4.154

[55] Rifon NJ Choi SM Trimble CS Li H. Congruence effects in sponsorship: the mediating role of sponsor credibility and consumer attributions of sponsor motive. J Advert. (2004) 33:30–42. 10.1080/00913367.2004.10639151

[56] Speed R Thompson P. Determinants of sports sponsorship response. J Acad Mark Sci. (2000) 28:226–38. 10.1177/0092070300282004

[57] Broniarczyk SM Alba JW. The importance of the brand in brand extension. J Mark Res. (1994) 31:214–28. 10.1177/002224379403100206

[58] Gwinner KP Eaton J. Building brand image through event sponsorship: the role of image transfer. J Advert. (1999) 28:47–57. 10.1080/00913367.1999.10673595

[59] Higgins ET. Making a good decision: value from Git. Am Psychol. (2000) 55:1217–30. 10.1037/0003-066X.55.11.1217 11280936

[60] Zdravkovic S Magusson P Stanley SM. Dimensions of fit between a brand and a social cause and their influence on attitudes. Int J Res Mark. (2010) 27:151–60. 10.1016/j.ijresmar.2010.01.005

[61] Lafferty BA. The relevance of fit in a cause–brand alliance when consumers evaluate corporate credibility. J Bus Res. (2007) 60:447–53. 10.1016/j.jbusres.2006.09.030

[62] Pierce AJ. Authentic identities. Soc Theory Pract. (2015) 41:435–57. 10.5840/soctheorpract201541323

[63] Varge S Guignon C. Authenticity. In: Zalta EN, editor. The Stanford Encyclopedia of Philosophy. Standford, CA: Standford University Press (2014). p. 1–10.

[64] Inoue Y Funk DC McDonald H. Predicting behavioral loyalty through corporate social responsibility: the mediating role of involvement and commitment. J Bus Res. (2017) 75:46–56. 10.1016/j.jbusres.2017.02.005

[65] Joo S Miller EG Fink JS. Consumer evaluations of CSR authenticity: development and validation of a multidimensional CSR authenticity scale. J Bus Res. (2019) 98:236–49. 10.1016/j.jbusres.2019.01.060

[66] Casper JM Pfahl ME McCullough BP. Is going green worth it? Assessing fan engagement and perceptions of athletic department environmental efforts. J Appl Sport Manag. (2017) 9:11. 10.18666/JASM-2017-V9-I1-7690

[67] Doherty AM Chen X Alexander N. The franchise relationship in China: agency and institutional theory perspectives. Eur J Mark. (2014) 48:1664–89. 10.1108/EJM-04-2012-0199

[68] Hu L-T Bentler PM. Cutoff criteria for fit indexes in covariance structure analysis: conventional criteria versus new alternatives. Struct Equ Model. (1999) 6:1–55. 10.1080/10705519909540118

[69] Hair JF Black B Babin B Anderson RE Tatham RL. Multivariate Data Analysis. 7th ed. New Jersey: Prentice-Hall (2009).

[70] Zinbarg RE Revelle W Yovel I Li W*.* Cronbach’s α, Revelle’s β, and McDonald’s ωH: their relations with each other and two alternative conceptualizations of reliability. Psychometrika. (2005) 70(1):123–33. 10.1007/s11336-003-0974-7

[71] Klein A Moosbrugger H. Maximum likelihood estimation of latent interaction effects with the LMS method. Psychometrika. (2000) 65:457–74. 10.1007/BF02296338

[72] Beaton AA Funk DC Ridinger L Jordan J. Sport involvement: a conceptual and empirical analysis. Sport Manag Rev. (2011) 14:126–40. 10.1016/j.smr.2010.07.002

[73] Ham S Han H. Role of perceived fit with hotels’ green practices in the formation of customer loyalty: impact of environmental concerns. Asia Pac J Tour Res. (2012) 18: 731–48. 10.1080/10941665.2012.695291

[74] Latif B Gunarathne N Gaskin J San Ong T Ali M. Environmental corporate social responsibility and pro-environmental behavior: the effect of green shared vision and personal ties. Resour Conserv Recycl. (2022) 186:106572. 10.1016/j.resconrec.2022.106572

[75] Chalip L Green BC Taks M Misener L. Creating sport participation from sport events: making it happen. Int J Sport Policy Polit. (2017) 9:257–76. 10.1080/19406940.2016.1257496

[76] Fredline E. Host and guest relations and sport tourism. Sport Soc*.* (2005) 8:263–79. 10.1080/17430430500087328

[77] Alexandris K Douka S Balaska P. Involvement with active leisure participation: does service quality have a role? Manag Leis. (2012) 17:54–66. 10.1080/13606719.2011.638203

[78] Kyle G Graefe A Manning R Bacon J. Predictors of behavioral loyalty among hikers along the Appalachian trail. Leis Sci. (2004) 26:99–118. 10.1080/01490400490272675

[79] Fornell C Larcker DF. Evaluating structural equation models with unobservable variables and measurement error. J Mark Res. (1981) 18:39–50. 10.2307/3151312

